# Shedding light into the black box of out-of-hospital respiratory distress—A retrospective cohort analysis of discharge diagnoses, prehospital diagnostic accuracy, and predictors of mortality

**DOI:** 10.1371/journal.pone.0271982

**Published:** 2022-08-03

**Authors:** Patrick Spörl, Stefan K. Beckers, Rolf Rossaint, Marc Felzen, Hanna Schröder

**Affiliations:** 1 Department of Anesthesiology, University Hospital RWTH Aachen, Aachen, Germany; 2 Aachen Institute for Rescue Management and Public Safety, University Hospital RWTH Aachen, Aachen, Germany; 3 Medical Direction, Emergency Medical Service, Aachen, Germany; University of Western Ontario, CANADA

## Abstract

**Background:**

Although respiratory distress is one of the most common complaints of patients requiring emergency medical services (EMS), there is a lack of evidence on important aspects.

**Objectives:**

Our study aims to determine the accuracy of EMS physician diagnostics in the out-of-hospital setting, identify examination findings that correlate with diagnoses, investigate hospital mortality, and identify mortality-associated predictors.

**Methods:**

This retrospective observational study examined EMS encounters between December 2015 and May 2016 in the city of Aachen, Germany, in which an EMS physician was present at the scene. Adult patients were included if the EMS physician initially detected dyspnea, low oxygen saturation, or pathological auscultation findings at the scene (n = 719). The analyses were performed by linking out-of-hospital data to hospital records and using binary logistic regressions.

**Results:**

The overall diagnostic accuracy was 69.9% (485/694). The highest diagnostic accuracies were observed in asthma (15/15; 100%), hypertensive crisis (28/33; 84.4%), and COPD exacerbation (114/138; 82.6%), lowest accuracies were observed in pneumonia (70/142; 49.3%), pulmonary embolism (8/18; 44.4%), and urinary tract infection (14/35; 40%). The overall hospital mortality rate was 13.8% (99/719). The highest hospital mortality rates were seen in pneumonia (44/142; 31%) and urinary tract infection (7/35; 20%). Identified risk factors for hospital mortality were metabolic acidosis in the initial blood gas analysis (odds ratio (OR) 11.84), the diagnosis of pneumonia (OR 3.22) reduced vigilance (OR 2.58), low oxygen saturation (OR 2.23), and increasing age (OR 1.03 by 1 year increase).

**Conclusions:**

Our data highlight the diagnostic uncertainties and high mortality in out-of-hospital emergency patients presenting with respiratory distress. Pneumonia was the most common and most frequently misdiagnosed cause and showed highest hospital mortality. The identified predictors could contribute to an early detection of patients at risk.

## Introduction

Emergency patients presenting with respiratory distress pose a major challenge to emergency medical service (EMS) professionals. On the one hand, available studies indicate that these patients have a markedly higher mortality rate than patients presenting with other complaints [1–3]. On the other hand, the high number of differential diagnoses and limited diagnostic options complicate accurate diagnosis in an out-of-hospital setting. Previous studies conducted in out-of-hospital settings [4–6] and emergency departments [2, 7, 8] have shown a wide range of possible causes of respiratory distress. This is a relevant issue because the available treatment algorithms are mostly based on the suspected underlying disease. An incorrect suspected diagnosis may result in the initiation of nonindicated procedures and a lack of indicated procedures, leading to a worse outcome [9]. Reliable out-of-hospital diagnosis is thus essential for effective out-of-hospital therapy.

But how accurate are EMS professionals in identifying the correct discharge diagnosis? To date, few studies have drawn conclusions about the diagnostic accuracy of EMS professionals in patients presenting with respiratory distress. Two previous studies highlight that the diagnostic error rates in these emergencies appear to be very high [10, 11]. However, detailed data on the diagnostic accuracy of specific diagnoses are often missing.

### Objectives

The primary goal of this study is to identify the underlying cause of each analyzed case to determine the diagnostic accuracy of EMS physicians in an out-of-hospital setting. In addition, it should be investigated which initial examination findings correlate with underlying causes. The second focus is to analyze the hospital mortality of this patient population to identify predictors of mortality, allowing for an early detection of patients at risk.

## Materials and methods

### Study design

This retrospective observational study was conducted within the EMS system in the city of Aachen, Germany, during a 6-month observation period (December 2015 to May 2016). All patients who presented with a respiratory problem and were treated by a physician-staffed emergency medical team (PEMT) were included in the analysis. The dataset was collected exclusively for the purpose of this study.

The study plan was submitted to the Ethics Committee of the Medical Faculty of RWTH Aachen University. In a statement dated July 9, 2018, the committee indicated that there were no ethical or legal objections to the study and specifically waived the need for ethics approval (file number EK 180/18). Data protection officers granted the use of the data and waived the requirement of informed consent.

### EMS system and operating area

The EMS in Germany is based on a two-tiered care system. An ambulance, manned by two paramedics, is dispatched after receiving an emergency call. The emergency dispatcher can also alert a PEMT staffed by an EMS physician and a paramedic. The PEMT is routinely alerted in potentially life-threatening cases (e.g., suspected cardiac arrest or severe respiratory failure). In addition, it can be requested by an ambulance whenever further medical support is needed at the scene.

Two 24/7 PEMTs, as well as 16 ambulances, serve the city of Aachen, which has approximately 250,000 inhabitants. All EMS physicians are experienced anesthesiologists, who are in at least the 4th year of their residency at the University Hospital RWTH Aachen. They must have spent at least one year in intensive care medicine and passed an 80-hour certifying course in emergency medicine. Three primary care hospitals and one university hospital were available for admission in the city of Aachen during the study period.

### Patient selection and data collection

**Fig 1:**
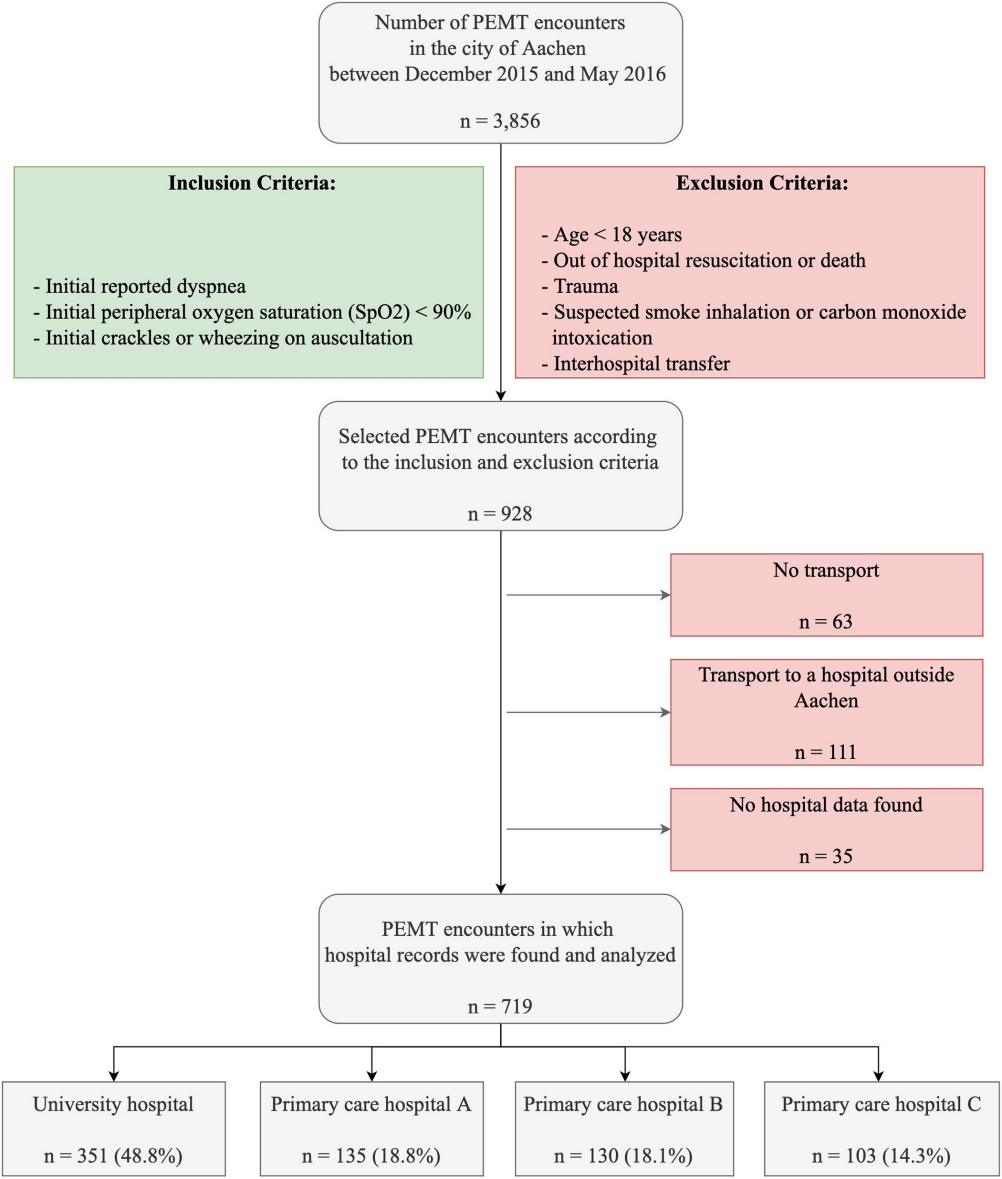
Study population considering the inclusion and exclusion criteria. **PEMT** physician-staffed emergency medical team.

We included all PEMT encounters in which patients were at least 18 years old and the EMS physician documented dyspnea, low oxygen saturation (SpO2 < 90%), or pathological auscultation findings initially at the scene. Fig 1 lists the inclusion and exclusion criteria we applied to identify adult nontraumatic respiratory emergencies.

Data acquisition was based on all items from the PEMT standardized protocols and was complemented with data from hospital records. The data from hospital records were manually collected and linked in accordance with data protection regulations. For this purpose, all four hospitals in the city of Aachen provided access to their hospital information systems. Data from the following sources were analyzed: handwritten PEMT standardized protocols, hospital discharge letters, and, if available, digital emergency department protocols as well as initial blood gas analyses.

### Terminology used in diagnoses

**Fig 2:**
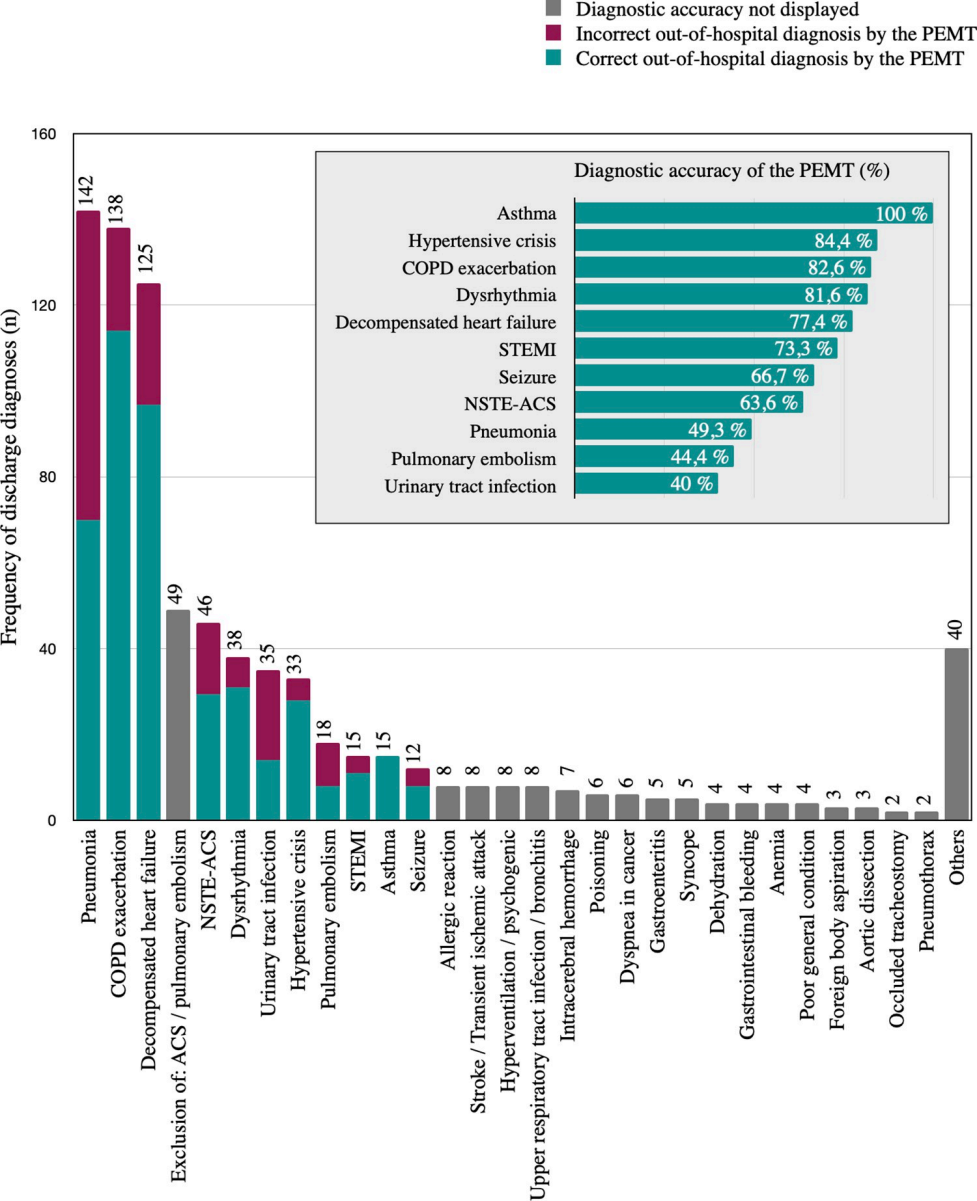
Frequency of discharge diagnoses and diagnostic accuracy of the PEMT. **PEMT:** physician-staffed emergency medical team; **COPD:** chronic obstructive pulmonary disease; **ACS:** acute coronary syndrome; **NSTE-ACS:** non-ST segment elevation ACS; **STEMI:** ST-elevation myocardial infarction; total of number of discharge diagnoses made: 793.

Diagnoses suspected by the PEMT are referred to as “out-of-hospital diagnoses” in this paper. The confirmed underlying cause for the emergency call, according to the hospital records, is referred to as “discharge diagnosis”. By reviewing the hospital data, we ensured that the diagnoses that resulted from complications during hospitalization were not considered discharge diagnoses. We standardized the terminology of the diagnoses by summarizing synonymous diseases. All discharge diagnoses were classified into one of those shown in Fig 2.

### Diagnostic accuracy of the PEMT

To assess diagnostic accuracy, we analyzed each hospital discharge letter and PEMT protocol and determined the consistency of the diagnoses. We considered each documented out-of-hospital diagnosis and defined it as correct if any of them matched the discharge diagnosis.

In deviation from this, we defined the out-of-hospital diagnoses of "suspected acute non-ST-segment elevation acute coronary syndrome" (NSTE-ACS) or "suspected pulmonary embolism" as correct if the only discharge diagnosis made was "exclusion of ACS" or "exclusion of pulmonary embolism." The authors believe that in these cases, out-of-hospital diagnoses cannot be considered incorrect because out-of-hospital exclusion is not possible with certainty and the PEMT did not miss other causes for the emergency call.

### Blood gas analysis (BGA)

All BGA results were obtained in the emergency departments (to date, blood gas analysis is not available in an out-of-hospital setting in the city of Aachen). It was often unclear whether BGA was performed with arterial, capillary, or venous blood. Therefore, we extended the reference ranges for pCO2, standard bicarbonate, and lactate to cover blood from all three sources. The reference ranges used are listed in S1 Table. Because the reference ranges for pO2 differ widely depending on the source of the blood, we did not consider pO2.

### Statistical analysis

Statistical analysis was performed using SPSS^®^ version 26 (IBM Corporation, Armonk, New York, USA). Two-sided p values <0.05 were considered significant.

## Identification of findings upon physical examination associated with discharge diagnoses

We used binary logistic regressions to test out-of-hospital and emergency department examination findings for associations with discharge diagnoses (all evaluated variables are listed below in Fig 4; for details, please refer to S2 Table). First, univariable analyses were made for each evaluated diagnosis. Multivariable analysis was performed in the second step to determine which pathological findings were independently associated with the respective discharge diagnoses. For this purpose, all variables with a p value < 0.20 in univariable analyses were considered. Due to the high number of missing data points for some variables (presented in S3 Table), the case count for the multivariable analyses was severely reduced. We considered the resulting risk of incorrect conclusions to be substantial and performed multiple imputations for all examination findings presented in S3 Table to minimize the risk of bias. The missing data were presumed to be missing at random, and 20 imputed datasets were generated using the fully conditional specification method based on the Markov chain Monte Carlo method. Multivariable analyses were performed on all imputed datasets, and the reported data represent the pooled results. Using multiple imputations significantly increased the number of cases included in the multivariable analyses (for example, from n = 243 to an average of n = 647 in the multivariable test for associations between examination findings and pneumonia).

### Predictors of hospital mortality

In Table 2 (and more detailed in S4 Table), we examine the association of predictors with hospital mortality using binary logistic regression. We analyzed non-BGA variables (listed in detail below Table 2) as well as the results of the initial blood gas analysis in the emergency department. All non-BGA variables were first tested using univariable analysis. In the second step, a multivariable analysis was performed to determine which pathologies were independently associated with hospital mortality. For this purpose, all non-BGA variables with a univariable p value < 0.20 were included. Because of the many missing data points for examination findings (presented in S3 Table), multiple imputations were performed, analogous to the procedure described above. Using imputed datasets increased the number of cases included in the multivariable analysis from n = 230 to an average of n = 620.

To assess the impact of BGA results on hospital mortality, we performed univariable analyses for all BGA results listed in S1 Table. BGA results with p < 0.2 in univariable analyses were tested in separate multivariable analyses which included all non-BGA variables with p < 0.2 in univariable analysis as well as the evaluated BGA variable.

## Results

In total, 928 PEMT encounters were considered according to the inclusion and exclusion criteria. These adult nontraumatic respiratory emergencies represented 24.1% of all PEMT encounters in the city of Aachen during the study period (n = 3,856). The study population consisted of 719 encounters in which hospital records were available.

### Characterization of the study population

Table 1 characterizes the patient population and presents information on PEMT encounters and hospital follow-up.

**Table 1:** Characterization of patients, PEMT encounters, and hospital follow-up.

Age (years), mean (standard deviation)	70.2 (16.7)
Female sex, n (%)	335 (46.9)
**PEMT alerting**	
Initial alerting on emergency call, n (%)	626 (87.1)
Alerting by ambulance on scene, n (%)	93 (12.9)
**Location of PEMT encounters**	
Flat, n (%)	493 (68.6)
Senior citizen’s home, n (%)	122 (17.0)
General practice, n (%)	34 (4.7)
Street, n (%)	24 (3.3)
Others, n (%)	46 (6.4)
**Frequency of out-of-hospital pathological findings**	
Dyspnea, n (%)	631 (87.8)
Wheezing upon auscultation, n (%)	188 (26.1)
Crackles upon auscultation, n (%)	181 (25.2)
Peripheral oxygen saturation < 90%, n (%)	308 (42.8)
**Hospital treatment**	
Outpatient treatment in the emergency department, n (%)	74 (10.3)
Inpatient hospital treatment, n (%)	645 (89.7)
ICU admission, n (%)	161 (22.4)
In-hospital death, n (%)	99 (13.8)
Length of stay in hospital (days), mean (standard deviation)	8.7 (12.5)

**PEMT** physician-staffed emergency medical team; **ICU** intensive care unit.

### Discharge diagnoses and diagnostic accuracy of the PEMT

A total of 793 discharge diagnoses were found. Hospital records defined one discharge diagnosis in 616 cases, two discharge diagnoses in 75 cases, and three discharge diagnoses in nine cases, while no reliable discharge diagnoses were made in 19 cases. Pneumonia (n = 142; 17.9%), COPD exacerbation (n = 138; 17.4%), and decompensated heart failure (n = 125; 15.8%) accounted for 51.1% (n = 405) of all discharge diagnoses. Fig 2 lists all discharge diagnoses made in our study and shows the diagnostic accuracy of the PEMT. Further descriptive data on demographics, physical examination findings, and hospitalization details for the most frequent discharge diagnoses are presented in S5 Table.

Overall, the diagnostic agreement between out-of-hospital diagnoses and discharge diagnoses was 69.9% (n = 485). Diagnostic accuracy could not be assessed in 25 of 719 encounters (3.5%) because either no out-of-hospital diagnosis was documented (n = 6) or the discharge diagnosis remained unclear (n = 19). Therefore, diagnostic accuracy analysis was performed on the remaining 694 encounters.

**Fig 3:**
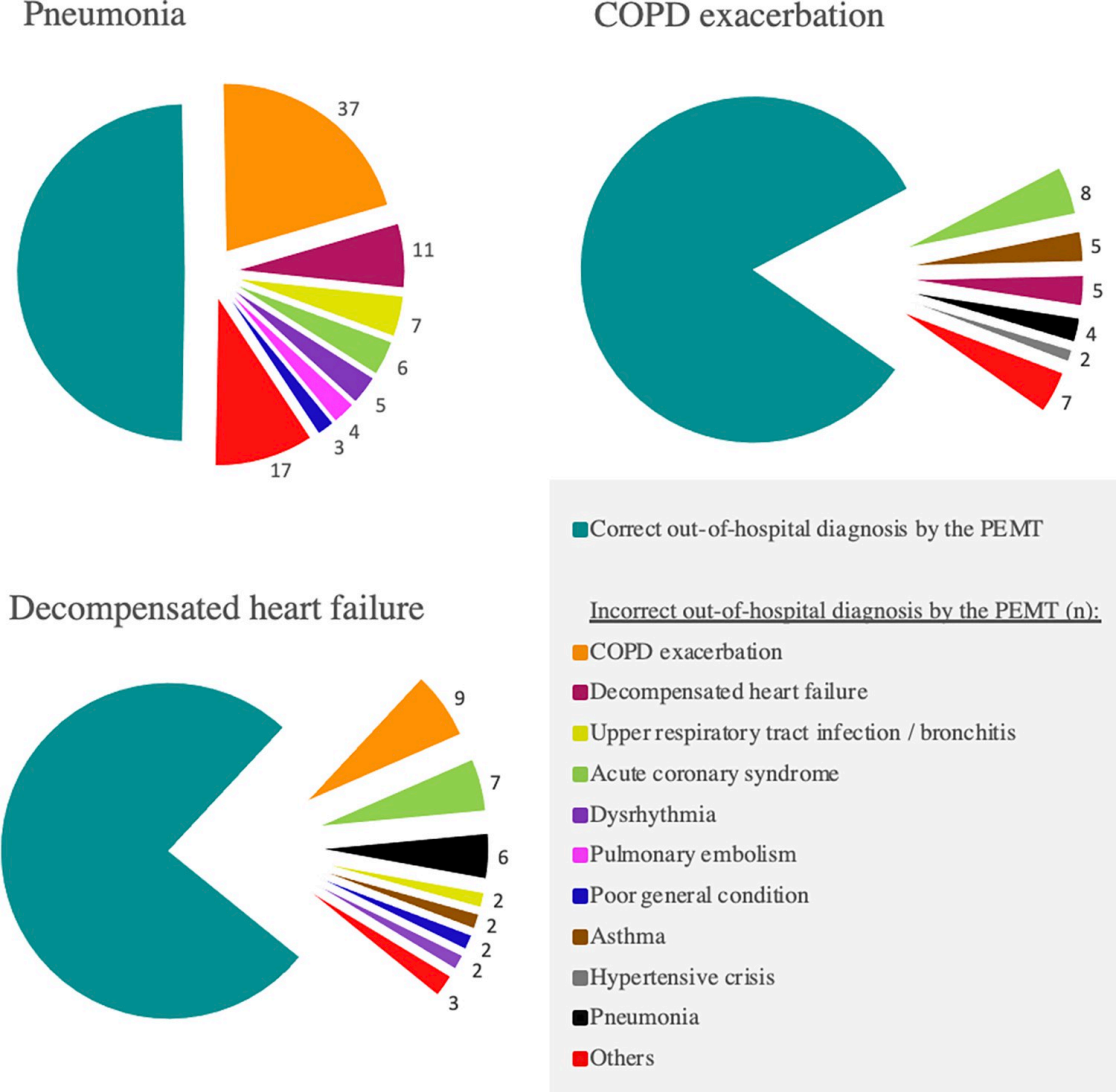
Which out-of-hospital diagnoses did the PEMTs suspect in cases of misdiagnosis? Fig 3 shows the incorrect PEMT out-of-hospital diagnoses for the three most frequent discharge diagnoses; **PEMT:** physician-staffed emergency medical team; **COPD:** chronic obstructive pulmonary disease.

Fig 3 shows which out-of-hospital diagnoses the PEMTs suspected when they failed to identify the correct discharge diagnosis.

### Characterization of discharge diagnoses and identification of associated findings upon physical examination

**Fig 4:**
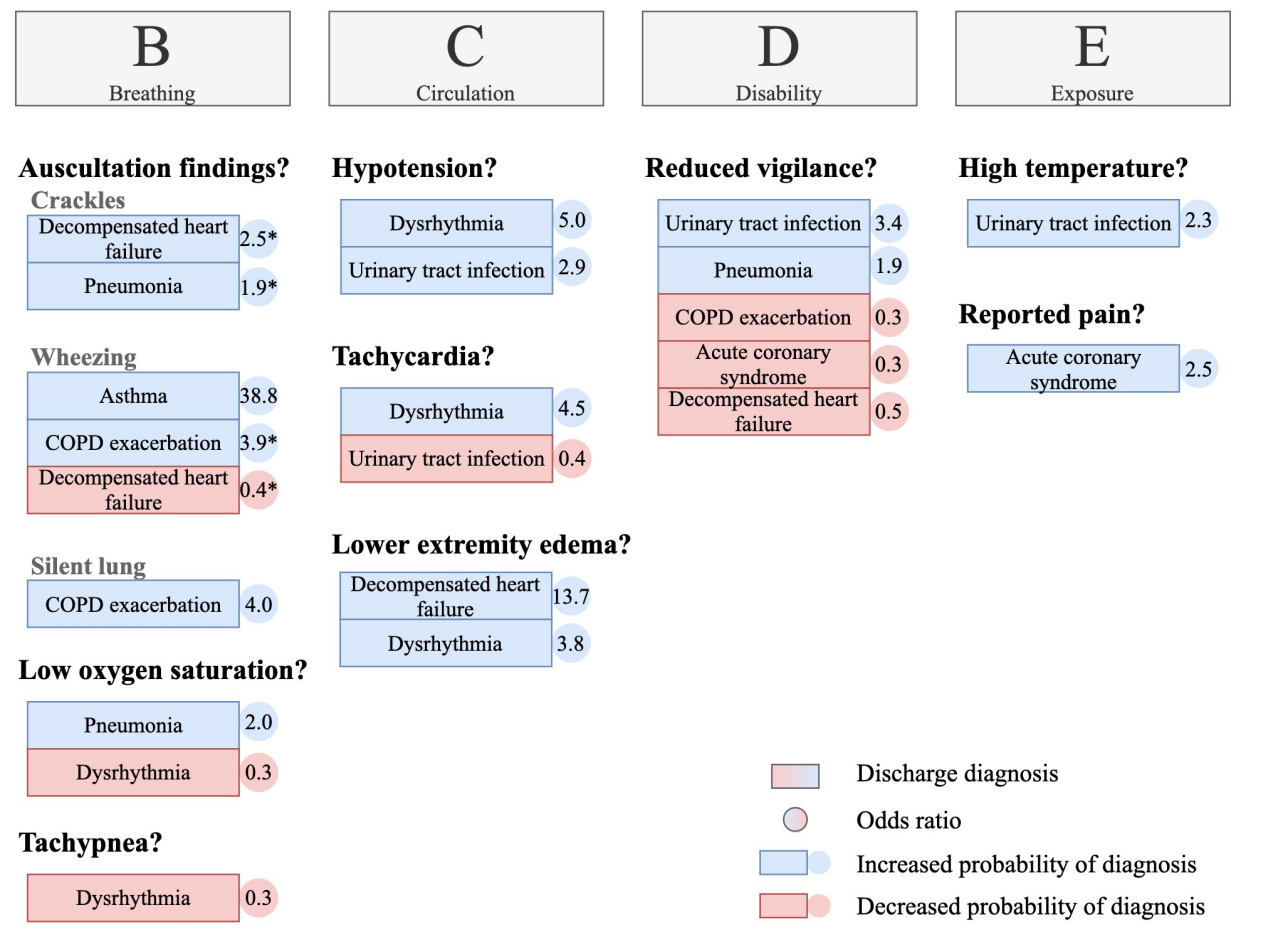
Associations of initial examination findings and discharge diagnoses. Binary logistic regression; only significant results are displayed, * mean of the odds ratios (when identical auscultation findings in the out-of-hospital setting and in the emergency department yielded significant results). The following variables were reviewed for associations with discharge diagnoses: out-of-hospital findings: hypotension (systolic blood pressure < 100 mmHg), tachycardia (heart rate > 100/min), low oxygen saturation (peripheral oxygen saturation < 90%), tachypnea (respiratory rate ≥ 22/min), high temperature (body temperature ≥ 38°C), body temperature ≤ 36°C, reduced vigilance (Glasgow Coma Scale < 15), reported pain (numeric rating scale ≥ 1), crackles upon auscultation, wheezing upon auscultation, emergency department findings: crackles upon auscultation, wheezing upon auscultation, silent lung upon auscultation, and lower extremity edema. Detailed results of logistic regressions are shown in S2 Table.

Fig 4 displays all out-of-hospital and emergency department examination findings that showed an independent association with the diagnoses evaluated. Detailed results of the statistical analysis are provided in S2 Table.

We found that some initial out-of-hospital examination findings were not documented (and probably not measured) in a relevant proportion of PEMT encounters (S3 Table). For example, respiratory rate was not documented in 26.1%, body temperature in 35.3%, and numeric rating scale in 39.9% of encounters.

### Hospital mortality and associated predictors

**Fig 5:**
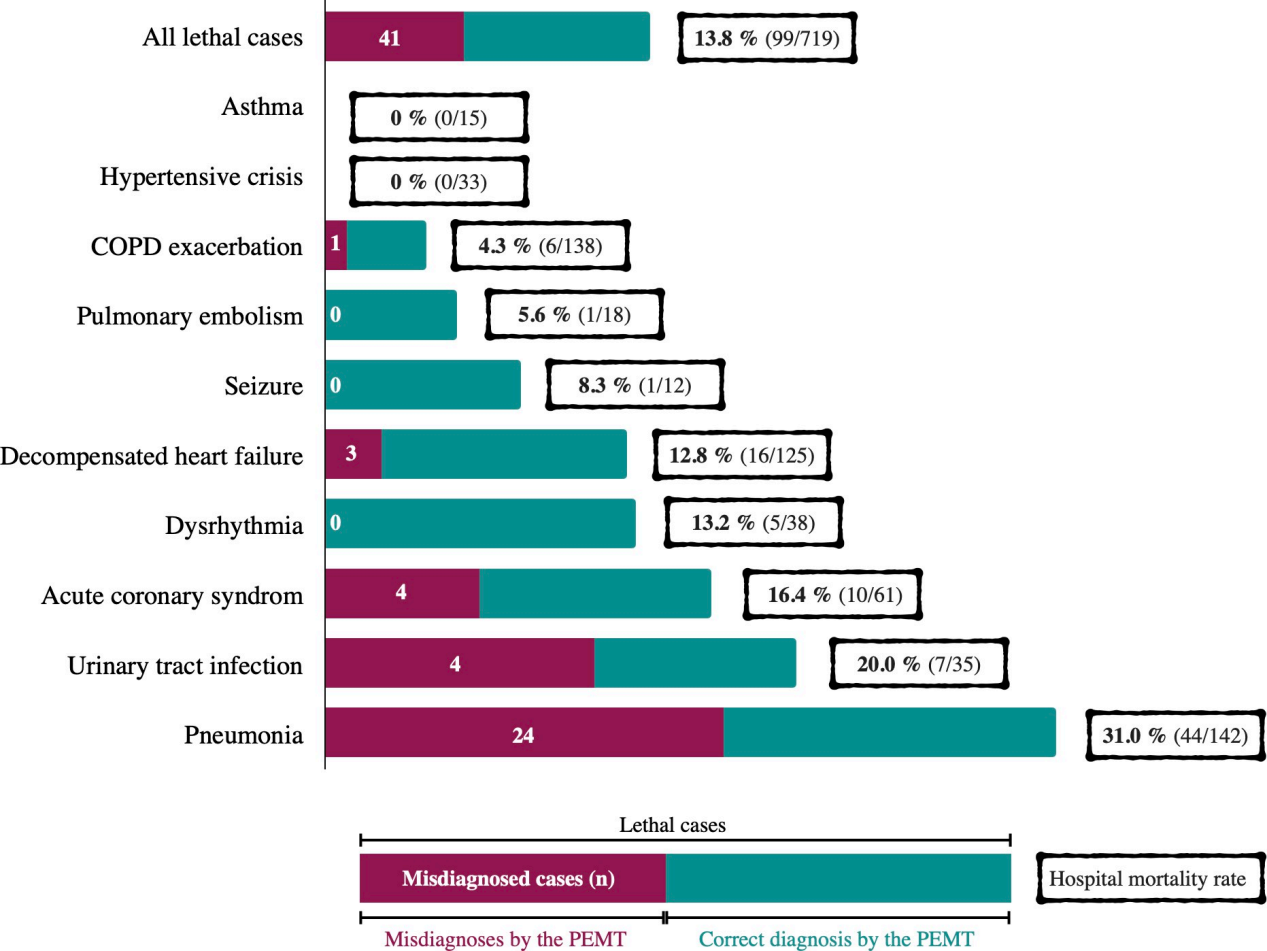
Hospital mortality and misdiagnoses by the PEMT. Fig 5 presents hospital mortality rates for the most frequent discharge diagnoses as well as the number of out-of-hospital misdiagnosed cases in lethal outcomes. **PEMT:** physician-staffed emergency medical team; **COPD:** chronic obstructive pulmonary disease.

The overall hospital mortality rate in our study was 13.8% (99/719). Fig 5 shows the hospital mortality rates of the most frequent discharge diagnoses and displays in how many of the lethal outcomes the PEMT made an incorrect out-of-hospital diagnosis.

In Table 2, we reviewed predictors for their association with hospital mortality. In multivariable analysis, reduced vigilance (GCS < 15), low oxygen saturation (SpO2 < 90%), and increasing age were associated with a higher risk of death, whereas wheezing upon auscultation was associated with lower mortality. The association between age and mortality was particularly evident for the 80- to 89-year-old group, while it was not seen for those over 89 years old (for further details see S4 Table). Moreover, the discharge diagnoses of pneumonia was associated with a significantly higher risk of hospital death.

**Table 2:** Predictors of hospital mortality.

	OR (95% CI)	p value
**Age**		
Age by 1 year increase	**1.031 (1.007–1.055)**	**0.011**
Age 80–89 years	**1.868 (1.033–3.378)**	**0.039**
**Pathological examination findings**		
Reduced vigilance (GCS < 15)	**2.583 (1.341–4.976)**	**0.005**
Low oxygen saturation (SpO2 < 90%)	**2.231 (1.193–4.173)**	**0.012**
Wheezing upon auscultation in the emergency department	**0.362 (0.136–0.961)**	**0.041**
**Discharge diagnoses**		
Pneumonia	**3.221 (1.562–6.644)**	**0.002**
**BGA results**		
pH < 7.35	**4.494 (1.740–11.608)**	**0.002**
pH < 7.30	**3.751 (1.346–10.457)**	**0.012**
Standard bicarbonate by 1 mmol/L increase	**0.903 (0.827–0.986)**	**0.023**
Standard bicarbonate < 17 mmol/L	**13.124 (1.970–87,415)**	**0.008**
Lactate by 1 mmol/L increase	**1.506 (1.072–2.117)**	**0.018**
Lactate > 4.0 mmol/L	**5.694 (1.066–30.423)**	**0.042**
Metabolic acidosis*	**11.841 (2.195–63.881)**	**0.004**

Binary logistic regression–results of multivariable analysis

First, univariable analyses were performed evaluating the following potential risk factors for hospital mortality: age, sex, discharge diagnoses with n > 10, out-of-hospital misdiagnosis by the PEMT, out-of-hospital findings: systolic blood pressure < 100 mmHg, heart rate > 100/min, peripheral oxygen saturation < 90%, respiratory rate ≥ 22/min, body temperature ≥ 38°C, body temperature ≤ 36°C, Glasgow Coma Scale < 15, numeric rating scale ≥ 1, crackles upon auscultation, wheezing upon auscultation, emergency department findings: crackles upon auscultation, wheezing upon auscultation, silent lung upon auscultation, lower extremity edema, and BGA results (listed in S1 Table).

Second, a multivariable analysis was performed including all non-BGA variables with p < 0.2 in univariable analyses.

All BGA result with p < 0.2 in univariable analyses were tested in separate multivariable analyses including all non-BGA variables with p < 0.2 in the univariable analysis. Table 2 lists all significant results in multivariable analyses (p < 0.05). Detailed results of logistic regressions are presented in S1 and S4 Tables.

**OR:** odds ratio; **95% CI:** 95% confidence interval of OR; **GCS:** Glasgow Coma Scale; **SpO2:** peripheral oxygen saturation; **pH:** hydrogen potential; **pCO2:** partial pressure of carbon dioxide; **HCO3:** standard bicarbonate; **OR:** odds ratio; **95% CI:** 95% confidence interval of OR; * Metabolic acidosis is defined as: pH < 7.35 and HCO3 < 21 mmol/L and pCO2 ≤ 6.7 kPa (50 mmHg) or pH < 7.35 and pCO2 ≤ 6.7 kPa (50 mmHg) and lactate > 5.0 mmol/L.

Meanwhile, an incorrect out-of-hospital diagnosis by the PEMT showed no significant effect on hospital mortality in multivariable analysis.

### Hospital mortality and initial blood gas analysis

Initial blood gas analyses were not performed in half of the cases. PH and pCO2 results were available in 364 of 719 cases (50.6%), and standard bicarbonate was available in 342 cases (47.6%). Because lactate measurement was exclusively measured at the participating university hospital, lactate results were available only in 192 cases (26.7%).

Analysis of the association between initial BGA and hospital mortality showed that metabolic acidosis significantly increased the risk of death. (Table 2). This association was evident in all BGA results related to metabolic acidosis (decreased pH, decreased standard bicarbonate, and increased lactate). More detailed results are shown in S1 Table.

## Discussion

This study of patients presenting with respiratory distress examined discharge diagnoses, the diagnostic accuracy of EMS physicians in an out-of-hospital setting, and hospital mortality. Our data show that diagnostic accuracy and hospital mortality differ widely depending on the discharge diagnoses. Overall, the high portions of misdiagnosis and the high hospital mortality rate confirm the assumption that this patient population seems to be particularly challenging and critically ill.

### Discharge diagnoses and diagnostic accuracy of the PEMT

The high proportion of 24.1% of all PEMT encounters during the study period shows the great relevance of nontraumatic respiratory emergencies in the daily routine of EMS. The highest prevalence was found for pneumonia, COPD exacerbation, and decompensated heart failure. This is largely consistent with the results of comparable out-of-hospital [5, 6] and emergency department [7, 8] studies. Seven other discharge diagnoses accounted for more than 25% of PEMT encounters in our study. This demonstrates that EMS professionals must consider a wide range of differential diagnoses when treating patients with respiratory distress.

Studies assessing the diagnostic accuracy of EMS professionals in respiratory emergencies are rare and differ in the way diagnostic accuracy was calculated.

In our study, the overall proportion of PEMT misdiagnoses was 30.1%. In two former PEMT studies from Germany, the highest portions for misdiagnosis (26% and 41%) were found in patients admitted for dyspnea [10, 11]. In contrast, two other German PEMT studies observed the highest misdiagnosis rates in neurological emergencies [12, 13]. Interestingly, these studies did not show a notable accumulation of misdiagnoses in respiratory emergencies. It is noticeable that one of them–the study by Arntz et al. [12]—differs in the qualifications of the EMS physicians involved. While 68% of the EMS physicians in this study were internists, only anesthesiologists worked as EMS physicians in all other studies (including ours). The strikingly low misdiagnosis rate described by Arntz et al. may be because internist-trained EMS physicians have more clinical experience in diagnosing patients with respiratory problems.

Looking at the individual discharge diagnoses in our study, there were huge differences in diagnostic accuracy. Low diagnostic accuracy is particularly notable for pneumonia, which was the most frequent discharge diagnosis in our study. Pneumonia was also associated with the highest hospital mortality rate (31%), the highest rate of ICU admissions (37.3%), and a significantly increased likelihood of in-hospital death. This observation is supported by two paramedic studies, which also found that patients with respiratory distress due to pneumonia had the highest mortality [5, 14]. Our analysis of PEMT misdiagnoses found that EMS physicians often suspected COPD exacerbation or decompensated heart failure when pneumonia was actually present.

It is less surprising that diagnostic accuracy was low for urinary tract infection. Presumably, few EMS professionals think primarily of a urinary tract infection in patients presenting with respiratory distress. However, patients admitted with urinary tract infection showed the second highest mortality rate of 20%. Closer examination of these cases revealed that a second discharge diagnosis (e.g., COPD exacerbation, pneumonia, or decompensated heart failure) was made in 12 of 35 patients admitted with a urinary tract infection. In these cases, it cannot be excluded that the respiratory symptoms were caused by these second discharge diagnoses. Urinary tract infections were at least concomitantly present in 4.9% of analyzed PEMT encounters and should therefore be considered a relevant differential diagnosis and cause for respiratory distress.

One former emergency department study of elderly patients with respiratory distress described that misdiagnosis resulted in worse patient outcomes [9]. In our study, the EMS physician made an incorrect diagnosis in 41.4% of all lethal cases. While there was a significant association between out-of-hospital misdiagnosis and in-hospital mortality in the univariable analysis, we did not observe it in the multivariable analysis. Nevertheless, it seems plausible that an increase in diagnostic accuracy would result in a decrease in morbidity and mortality.

### Pathological findings associated with discharge diagnoses

The two strongest associations we found were of prehospital wheezing being strongly associated with asthma (odds ratio 38.8) and lower extremity edema being associated with decompensated heart failure (odds ratio 13.7). Our data indicate that a clear recommendation can be made for thorough auscultation of the lungs and evaluation of lower extremity edema in all patients with respiratory distress.

Several findings correlated with low or moderate odds ratios with discharge diagnoses. It became clear that many routine parameters (e.g., body temperature and GCS) provide important information about underlying diagnoses. Nevertheless, some of these parameters were not documented in many encounters.

Our data indicate that a thorough and complete physical examination, as well as consideration of the numerous differential diagnoses are requirements for making a reliable out-of-hospital diagnosis, especially in cases of respiratory distress. This even allows for the identification of unexpected pathological findings (e.g., high body temperature), which can provide essential hints for the correct diagnosis.

In our opinion, the prehospital established ABCDE approach (airway, breathing, circulation, disability, environment) is suitable for making reliable diagnoses even in patients with respiratory distress, as long as followed conscientiously. In this regard, EMS professionals should be sensitized that a thorough search, especially for B and C problems, is essential for a correct diagnosis. Moreover, focused education and training—particularly in distinguishing the causes of respiratory distress—could help ensure that EMS professionals are better prepared to deal with these difficult emergencies in the future.

Several emergency department studies have shown that the use of point-of-care ultrasound (POCUS) in respiratory emergencies can reduce the number of differential diagnoses and increase diagnostic accuracy [15–17]. Out-of-hospital use of ultrasound and training of EMS professionals in POCUS therefore have the potential to increase diagnostic accuracy.

### Hospital mortality and the search for associated predictors

The hospital mortality rate in our study was 13.8%, which was comparable to that of four comparable EMS studies involving patients with dyspnea [1, 4, 5, 14]. These studies reported hospital or 30-day mortality rates from 11% to 13.2%. We attribute the slightly higher mortality in our study to the fact that only EMS encounters involving EMS physicians were analyzed and that these encounters presumably represent a particularly critical subset of all respiratory emergencies. Unsurprisingly, the ICU admission rate in our study was also very high (22.4%). The consistent above-average mortality and ICU admission rates highlight that patients with respiratory distress should be considered high-risk.

According to our analysis, decreased vigilance, low oxygen saturation, and increasing age can be considered independent predictors for hospital mortality. Two former emergency department studies that included patients with any complaint also found that decreased vigilance was an independent risk factor for 30-day mortality [3, 18]. The same conclusion was made in an EMS study of patients with the complaint of dyspnea [19]. Two of these studies [3, 19] similarly reported that low oxygen saturation was a risk factor for mortality. Patients with dyspnea have a higher risk of death with increasing age, which has also been shown by previous studies [9, 19].

### Hospital mortality and initial blood gas analysis

Examination of the initial BGA results shows that metabolic acidosis appears to be a strong risk factor for death.

Surprisingly, there is little evidence of the prognostic value of BGA results in patients with respiratory distress. Two previous studies showed that acidosis led to a higher risk of mortality or ICU admission in patients with dyspnea [20, 21]. Numerous studies have demonstrated that initial hyperlactatemia is an independent predictor of mortality in unselected emergency department patients [22–24]. Our results highlight that elevated lactate is an important prognostic parameter even in emergency patients with respiratory distress. Therefore, BGA, including lactate, should be routinely measured in all patients presenting with respiratory distress.

### Limitations

Compared to other studies, our study is based on a relatively small sample size. Because we examined only PEMT encounters during winter and spring, our results do not allow for conclusions about all respiratory emergencies throughout an entire year. Rather, it can be assumed that we studied a particularly critically ill subset of this patient population. Since the evaluation of diagnostic accuracy was not blinded, it cannot be excluded with certainty that it was over- or underestimated. The many missing data in some examination findings posed challenges for the multivariable analyses. We tried to compensate them by running multiple imputations. The sample size for BGA analysis was particularly small, as BGA results were obtained from only half and lactate was measured in a quarter of the encounters. Because it often remained unclear whether venous, capillary, or arterial blood samples were analyzed, it was not reasonable to consider pO2 (as the respective reference ranges differ widely).

## Conclusions

We showed that the overall diagnostic uncertainty in patients with respiratory distress was high but varied greatly between discharge diagnoses. Misdiagnoses occurred particularly frequently in patients with pneumonia. At the same time, pneumonia was responsible for most EMS encounters, most ICU admissions, and most hospital deaths.

Analysis of hospital mortality showed that emergency patients presented with respiratory distress had a markedly high risk of in-hospital death. According to our data, decreased vigilance, low oxygen saturation, increasing age, and metabolic acidosis can be considered risk factors for hospital mortality.

Study findings call for a thorough physical examination and consideration of the many differential diagnoses to increase diagnostic accuracy in the future.

## Supporting information

S1 Table Associations between initial blood gas analysis in the emergency department and hospital mortality. **Binary logistic regression:** All BGA results for which univariable analyses were performed are displayed. Variables included in multivariable analysis are: age by 1 year, sex and examination findings with p < 0.2 in univariable analysis (see S4 Table). Significant findings (p < 0.05) are shaded grey. **Reference range:** adjusted for blood from arterial, capillary, and venous source; *pathological findings are defined as follows: **Respiratory acidosis:** pH < 7.35 and pCO2 > 6.7 kPa (50 mmHg) and standard bicarbonate (HCO3) ≥ 21 mmol/L; **Respiratory alkalosis:** pH > 7.45 and pCO2 < 4.7 kPa (35 mmHg) and HCO3 ≤ 30 mmol/L; **Metabolic acidosis:** (pH < 7.35 and HCO3 < 21 mmol/L and pCO2 ≤ 6.7 kPa (50 mmHg)) or (pH < 7.35 and pCO2 ≤ 6.7 kPa (50 mmHg) and lactate > 5.0 mmol/L); **Lactate acidosis:** pH < 7.35 and pCO2 ≤ 6.7 kPa (50 mmHg) and lactate > 5.0 mmol/L; **Metabolic acidosis of a cause other than lactate:** pH < 7.35 and HCO3 < 21 mmol/L and pCO2 ≤ 6.7 kPa (50 mmHg) and lactate ≤ 2.2. mmol/L; **Metabolic alkalosis:** pH < 7.35 and HCO3 < 21 mmol/L and pCO2 ≤ 6.7 kPa (50 mmHg).
(DOCX)

S2 Table Association between initial examination findings and most frequent discharge diagnoses. Binary logistic regression: findings with p < 0.2 in univariable analysis are shown and included in multivariable analysis; significant findings (p < 0.05) are shaded grey. The following variables were reviewed for associations with discharge diagnoses: out-of-hospital findings: systolic blood pressure < 100 mmHg, heart rate > 100/min, peripheral oxygen saturation < 90%, respiratory rate ≥ 22/min, body temperature ≥ 38°C, body temperature ≤ 36°C, Glasgow Coma Scale < 15, numeric rating scale ≥ 1, crackles upon auscultation, wheezing upon auscultation, emergency department findings: crackles upon auscultation, wheezing upon auscultation, silent lung upon auscultation, and lower extremity edema.
(DOCX)

S3 Table Parameters not documented by the PEMT. Proportion of initial out-of-hospital examination findings that were not documented in the PEMT protocol.
(DOCX)

S4 Table Predictors of hospital mortality. **Binary logistic regression:** the following variables were tested for their associations with hospital mortality using univariable analyses: age, sex, misdiagnosis by the PEMT, discharge diagnoses with n > 10, out-of-hospital findings: systolic blood pressure < 100 mmHg, heart rate > 100/min, peripheral oxygen saturation < 90%, respiratory rate ≥ 22/min, body temperature ≥ 38°C, body temperature ≤ 36°C, Glasgow Coma Scale < 15, numeric rating scale ≥ 1, crackles upon auscultation, wheezing upon auscultation, emergency department findings: crackles upon auscultation, wheezing upon auscultation, silent lung upon auscultation, and lower extremity edema. Any variables with p < 0.2 in the univariable analysis are shown in the table and included in multivariable analysis. Significant findings (p < 0.05) are shaded grey.
(DOCX)

S5 Table Characterization of the most frequent discharge diagnoses. (DOCX)

S1 Dataset Data set used for statistical analysis. (XLSX)

## Abbreviations

ACS: acute coronary syndrome
BGA: blood gas analysis
CI: confidence interval
COPD: chronic obstructive pulmonary disease
EMS: emergency medical service
GCS: Glasgow Coma Scale
HCO3: standard bicarbonate
ICU: intensive care unit
NRS: numeric rating scale
NSTE-ACS: non-ST segment elevation acute coronary syndrome
OR: odds ratio
pCO2: partial pressure of carbon dioxide
pH: hydrogen potential
POCUS: point-of-care ultrasound
PEMT: physician-staffed emergency medical team
pO2: partial pressure of oxygen
SpO2: peripheral oxygen saturation
STEMI: ST-elevation myocardial infarction
