# Supplementary material for: Shedding light into the black box of out-of-hospital respiratory distress—A retrospective cohort analysis of discharge diagnoses, prehospital diagnostic accuracy, and predictors of mortality
Source: PLoS One. 2022 Aug 3;17(8):e0271982. doi: 10.1371/journal.pone.0271982 (PMC9348717; doi:10.1371/journal.pone.0271982)
Supplement: S1 Table — Binary logistic regression: All BGA results for which univariable analyses were performed are displayed. Variables included in multivariable analysis are: age by 1 year, sex and examination findings with p < 0.2 in univariable analysis (see S4 Table). Significant findings (p < 0.05) are shaded grey. Reference range: adjusted for blood from arterial, capillary, and venous source; *pathological findings are defined as follows: Respiratory acidosis: pH < 7.35 and pCO2 > 6.7 kPa (50 mmHg) and standard bicarbonate (HCO3) ≥ 21 mmol/L; Respiratory alkalosis: pH > 7.45 and pCO2 < 4.7 kPa (35 mmHg) and HCO3 ≤ 30 mmol/L; Metabolic acidosis: (pH < 7.35 and HCO3 < 21 mmol/L and pCO2 ≤ 6.7 kPa (50 mmHg)) or (pH < 7.35 and pCO2 ≤ 6.7 kPa (50 mmHg) and lactate > 5.0 mmol/L); Lactate acidosis: pH < 7.35 and pCO2 ≤ 6.7 kPa (50 mmHg) and lactate > 5.0 mmol/L; Metabolic acidosis of a cause other than lactate: pH < 7.35 and HCO3 < 21 mmol/L and pCO2 ≤ 6.7 kPa (50 mmHg) and lactate ≤ 2.2. mmol/L; Metabolic alkalosis: pH < 7.35 and HCO3 < 21 mmol/L and pCO2 ≤ 6.7 kPa (50 mmHg). (DOCX) [file pone.0271982.s001.docx]

**S1 Table. Associations between initial blood gas analysis in the emergency department and hospital mortality.**

|  | **Present/**  **encounters in which data is available n/n (%)** | **Present/**  **deaths in which data is available n/n (%)** | **Univariable analysis** | | **Multivariable analysis using multiple imputations** | |
| --- | --- | --- | --- | --- | --- | --- |
|  |  |  | **OR (95% CI)** | **p value** | **OR (95% CI)** | **p value** |
| **pH value by 1 unit increase** |  |  | 0.728 (0.339-1.565) | 0.416 |  |  |
| pH > 7.50 | 13/364 (3.6) | 1/44 (2.3) | 0.597 (0.076-4.706) | 0.624 |  |  |
| pH > 7.45 | 66/364 (18.1) | 5/44 (11.4) | 0.544 (0.206-1.439) | 0.220 |  |  |
| pH ≥ 7.35 ≤ 7.45 - reference range - | 176/364 (48.4) | 14/44 (34.1) | **0.511 (0.264-0.989)** | **0.046** | 0.596 (0.250-1.420) | 0.242 |
| pH < 7.35 | 122/364 (33.5) | 24/44 (54.5) | **2.718 (1.434-5.151)** | **0.002** | **4.494 (1.740-11.608)** | **0.002** |
| pH < 7.3 | 65/364 (17.9) | 16/44 (36.4) | **3.160 (1.592-6.272)** | **0.001** | **3.751 (1.346-10.457)** | **0.012** |
| pH < 7.25 | 39/364 (10.7) | 10/44 (22.7) | **2.951 (1.324-6.580)** | **0.008** | 3.026 (0.968-9.457) | 0.057 |
| pH < 7.2 | 21/364 (5.8) | 6/44 (13.6) | **3.211 (1.175-8.771)** | **0.023** | 2.649 (0.653-10.751) | 0.173 |
| **pCO2 by 1 mmHg increase** |  |  | 1.000 (0.980-1.020) | 0.994 |  |  |
| pCO2 < 3,6 kPa (27 mmHg) | 12/364 (3.3) | 4/43 (9.3) | **4.013 (1.155-13.944)** | **0.029** | 2.748 (0.392-19.286) | 0.309 |
| pCO2 < 4.0 kPa (30 mmHg) | 28/364 (7.7) | 6/43 (14.0) | 2.204 (0.840-5.786) | 0.109 | 1.493 (0.388-5.735) | 0.559 |
| pCO2< 4.7 kPa (35 mmHg) | 74/364 (20.3) | 7/43 (16.3) | 0.737 (0.314-1.730) | 0.484 |  |  |
| pCO2 ≥ 4.7 ≤ 6.7 kPa (≥ 35 ≤ 50 mmHg)  - reference range - | 174/364 (47.8) | 20/43 (46.5) | 0.943 (0.498-1.785) | 0.857 |  |  |
| pCO2 > 6.7 kPa (50 mmHg) | 116/364 (31.9) | 16/43 (37.2) | 1.310 (0.676-2.539) | 0.424 |  |  |
| pCO2 > 8.0 kPa (60 mmHg) | 62/364 (17.0) | 7/43 (16.3) | 0.940 (0.398-2.223) | 0.889 |  |  |
| pCO2 > 9.3 kPa (70 mmHg) | 34/364 (9.3) | 4/43 (9.3) | 0.995 (0.333-2.975) | 0.993 |  |  |
| pCO2 > 10.7 kPa (80 mmHg) | 20/364 (5.5) | 3/43 (7.0) | 1.341 (0.376-4.780) | 0.651 |  |  |
| **Standard bicarbonate by 1 mmol/L increase** |  |  | **0.895 (0.839-0.954)** | **0.001** | **0.903 (0.827-0.986)** | **0.023** |
| HCO3 < 17 mmol/L | 10/342 (2.9) | 7/43 (16.3) | **19.185 (4.750-77.493)** | **<0.001** | **13.124 (1.970-87,415)** | **0.008** |
| HCO3 < 21 mmol/L | 40/342 (11.7) | 12/43 (27.9) | **3.747 (1.732-8.105)** | **0.001** | 2.310 (0.743-7.181) | 0.148 |
| HCO3 ≥ 21 ≤ 30 mmol/L - reference range - | 217/342 (63.5) | 22/43 (51.2) | 0.559 (0.294-1.063) | 0.076 | 0.742 (0.305-1.805) | 0.510 |
| HCO3 > 30 mmol/L | 85/342 (24.9) | 9/43 (20.9) | 0.777 (0.356-1.693) | 0.525 |  |  |
| HCO3 > 34 mmol/L | 35/342 (10.2) | 4/43 (9.3) | 0.887 (0.297-2.648) | 0.829 |  |  |
| HCO3 > 38 mmol/L | 14/342 (4.1) | 1/43 (2.3) | 0.524 (0.067-4.108) | 0.538 |  |  |
| **Lactate by 1 mmol/L increase** |  |  | **1.646 (1.251-2.165)** | **<0.001** | **1.506 (1.072-2.117)** | **0.018** |
| Lactate ≤ 2.2 mmol/L - reference range - | 129/192 (67.2) | 11/27 (40.7) | **0.274 (0.118-0.634)** | **0.002** | 0.424 (0.127-1.414) | 0.162 |
| Lactate > 2.2 mmol/L | 63/192 (32.8) | 16/27 (59.3) | **3.652 (1.578-8.449)** | **0.002** | 2.359 (0.707-7.867) | 0.162 |
| Lactate > 3.0 mmol/L | 31/192 (16.1) | 10/27 (37.0) | **4.034 (1.631-9.975)** | **0.003** | 2.228 (0.569-8.727) | 0.250 |
| Lactate > 4.0 mmol/L | 19/192 (9.9) | 9/27 (33.3) | **7.750 (2.783-21.585)** | **<0.001** | **5.694 (1.066-30.423)** | **0.042** |
| Lactate > 5.0 mmol/L | 10/192 (5.2) | 5/27 (18.5) | **7.273 (1.948-27.151)** | **0.003** | 5.189 (0.439-61.258) | 0.190 |
| **Pathological findings*:** | 150/342 (43.9) | 23/43 (53.5) |  |  |  |  |
| Respiratory acidosis | 75/342 (21.9) | 10/43 (23.3) | 0.822 (0.511-2.330) | 0.822 |  |  |
| Respiratory alkalosis | 37/342 (10.8) | 3/43 (7.0) | 0.585 (0.171-1.993) | 0.391 |  |  |
| Metabolic acidosis | 16/342 (4.7) | 8/43 (18.6) | **8.314 (2.936-23.542)** | **<0.001** | **11.841 (2.195-63.881)** | **0.004** |
| Metabolic alkalosis | 22/342 (6.4) | 2/43 (4.7) | 0.680 (0.153-3.020) | 0.613 |  |  |

***Binary logistic regression:*** *All BGA results for which univariable analyses were performed are displayed. Variables included in multivariable analysis are: age by 1 year, sex and examination findings with p < 0.2 in univariable analysis (see S4 Table). Significant findings (p < 0.05)* *are shaded grey.*

***Reference range:*** *adjusted for blood from arterial, capillary, and venous source;* ***pH:*** *hydrogen potential;* ***pCO2:*** *partial pressure of carbon dioxide;* ***HCO3:*** *standard bicarbonate;* ***OR:*** *odds ratio;* ***95% CI:*** *95% confidence interval of OR;* ********pathological findings are defined as follows:* ***Respiratory acidosis:*** *pH < 7.35 and pCO2 > 6.7 kPa (50 mmHg) and standard bicarbonate (HCO3) ≥ 21 mmol/L;* ***Respiratory alkalosis:*** *pH > 7.45 and pCO2 < 4.7 kPa (35 mmHg) and HCO3 ≤ 30 mmol/L;* ***Metabolic acidosis:*** *(pH < 7.35 and HCO3 < 21 mmol/L and pCO2 ≤ 6.7 kPa (50 mmHg)) or (pH < 7.35 and pCO2 ≤ 6.7 kPa (50 mmHg) and lactate > 5.0 mmol/L);* ***Lactate acidosis:*** *pH < 7.35 and pCO2 ≤ 6.7 kPa (50 mmHg) and lactate > 5.0 mmol/L;* ***Metabolic acidosis of a cause other than lactate:*** *pH < 7.35 and HCO3 < 21 mmol/L and pCO2 ≤ 6.7 kPa (50 mmHg) and lactate ≤ 2.2. mmol/L;* ***Metabolic alkalosis:*** *pH < 7.35 and HCO3 < 21 mmol/L and pCO2 ≤ 6.7 kPa (50 mmHg).*
