# Supplementary material for: Shedding light into the black box of out-of-hospital respiratory distress—A retrospective cohort analysis of discharge diagnoses, prehospital diagnostic accuracy, and predictors of mortality
Source: PLoS One. 2022 Aug 3;17(8):e0271982. doi: 10.1371/journal.pone.0271982 (PMC9348717; doi:10.1371/journal.pone.0271982)
Supplement: S2 Table — Binary logistic regression: findings with p < 0.2 in univariable analysis are shown and included in multivariable analysis; significant findings (p < 0.05) are shaded grey. The following variables were reviewed for associations with discharge diagnoses: out-of-hospital findings: systolic blood pressure < 100 mmHg, heart rate > 100/min, peripheral oxygen saturation < 90%, respiratory rate ≥ 22/min, body temperature ≥ 38°C, body temperature ≤ 36°C, Glasgow Coma Scale < 15, numeric rating scale ≥ 1, crackles upon auscultation, wheezing upon auscultation, emergency department findings: crackles upon auscultation, wheezing upon auscultation, silent lung upon auscultation, and lower extremity edema. (DOCX) [file pone.0271982.s002.docx]

**S2 Table. Association between initial examination findings and most frequent discharge diagnoses.**

| **Discharge diagnoses and**  **pathological findings** | **Present/**  **available data n/n (%)** | **Univariable analysis** | | **Multivariable analysis using multiple imputations** | |
| --- | --- | --- | --- | --- | --- |
|  |  | **OR (95% CI)** | **p value** | **OR (95% CI)** | **p value** |
| **Pneumonia (n = 142)** | | | | | |
| Low oxygen saturation (SpO2 < 90%) | 94/142 (66.2) | **3.252 (2.209-4.787)** | **<0.001** | **1.993 (1.231-3.228)** | **0.005** |
| Crackles upon auscultation (out-of-hospital) | 65/142 (45.8) | **3.355 (2.276-4.944)** | **<0.001** | **1.952 (1.186-3.213)** | **0.008** |
| Reduced vigilance (GCS < 15) | 52/137 (38.0) | **2.801 (1.866-4.204)** | **<0.001** | **1.904 (1.162-3.120)** | **0.011** |
| Crackles upon auscultation (emergency department) | 52/142 (36.6) | **2.963 (1.973-4.449)** | **<0.001** | **1.896 (1.124-3.200)** | **0.017** |
| High body temperature (temperature ≥ 38 °C) | 38/118 (32.2) | **3.032 (1.851-4.967)** | **<0.001** | 1.698 (0.976-2.955) | 0.061 |
| Tachycardia (heart rate > 100/minute) | 78/139 (56.1) | **1.981 (1.362-2.881)** | **<0.001** | 1.547 (0.984-2.433) | 0.059 |
| Tachypnea (respiratory rate ≥ 22/minute) | 56/111 (50.5) | **1.952 (1.278-2.979)** | **0.002** | 1.386 (0.855-2.247) | 0.185 |
| Reported pain (NRS ≥ 1) | 12/81 (14.8) | **0.296 (0.154-0.566)** | **<0.001** | 0.728 (0.466-1.136) | 0.162 |
| Lower extremity edema (emergency department) | 15/142 (10.6) | 0.674 (0.377-1.207) | 0.185 | 0.661 (0.334-1.307) | 0.234 |
| **COPD exacerbation (n = 138)** | | | | | |
| Wheezing upon auscultation (emergency department) | 81/138 (58.7) | **7.441 (4.963-11.157)** | **<0.001** | **4.738 (2.916-7.698)** | **<0.001** |
| Silent lung upon auscultation (emergency department) | 20/138 (14.5) | **4.105 (2.183-7.717)** | **<0.001** | **4.062 (1.796-9.186)** | **0.001** |
| Wheezing upon auscultation (out-of-hospital) | 79/138 (57.2) | **5.798 (3.901-8.619)** | **<0.001** | **3.002 (1.779-5.006)** | **<0.001** |
| Low oxygen saturation (SpO2 < 90%) | 71/138 (51.4) | **1.505 (1.037-2.185)** | **0.032** | 1.499 (0.886-2.536) | 0.131 |
| Tachycardia (heart rate > 100/minute) | 71/137 (51.8) | **1.593 (1.095-2.316)** | **0.015** | 1.420 (0.867-2.327) | 0.163 |
| Tachypnea (respiratory rate ≥ 22/minute) | 55/111 (49.5) | **1.863 (1.220-2.843)** | **0.004** | 1.010 (0.572-1.785) | 0.972 |
| High body temperature (temperature ≥ 38 °C) | 14/104 (13.5) | 0.635 (0.342-1.181) | 0.152 | 0.988 (0.514-1.898) | 0.970 |
| Reported pain (NRS ≥ 1) | 16/89 (18.0) | **0.377 (0.211-0.677)** | **0.001** | 0.700 (0.422-1.159) | 0.165 |
| Crackles upon auscultation (out-of-hospital) | 27/138 (19.6) | 0.674 (0.426-1.067) | 0.093 | 0.574 (0.295-1.117) | 0.102 |
| Hypotension (systolic blood pressure < 100 mmHg) | 3/134 (2.2) | **0.258 (0.079-0.842)** | **0.025** | 0.527 (0.118-2.354) | 0.402 |
| Reduced vigilance (GCS < 15) | 7/135 (5.2) | **0.158 (0.072-0.345)** | **<0.001** | **0.279 (0.113-0.690)** | **0.006** |
| **Decompensated heart failure (n = 125)** | | | | | |
| Lower extremity edema (emergency department) | 62/125 (49.6) | **14.005 (8.685-22.585)** | **<0.001** | **13.686 (8.041-23.295)** | **<0.001** |
| Crackles upon auscultation (emergency department) | 53/125 (42.4) | **4.021 (2.645-6.114)** | **<0.001** | **3.010 (1.792-5.057)** | **<0.001** |
| Crackles upon auscultation (out-of-hospital) | 49/125 (39.2) | **2.257 (1.501-3.393)** | **<0.001** | **1.922 (1.119-3.301)** | **0.018** |
| Low oxygen saturation (SpO2 < 90%) | 67/125 (53.6) | **1.656 (1.124-2.441)** | **0.011** | 1.604 (0.980-2.625) | 0.060 |
| Tachycardia (heart rate > 100/minute) | 40/120 (33.3) | **0.626 (0.414-0.946)** | **0.026** | 0.728 (0.448-1.184) | 0.201 |
| Reduced vigilance (GCS < 15) | 18/123 (14.6) | **0.563 (0.330-0.963)** | **0.036** | **0.464 (0.247-0.869)** | **0.016** |
| Wheezing upon auscultation (emergency department) | 21/125 (16.8) | **0.588 (0.355-0.973)** | **0.039** | **0.335 (0.180-0.624)** | **0.001** |
| **Acute coronary syndrome (n = 61)** | | | | | |
| Reported pain (NRS ≥ 1) | 29/42 (69) | **5.468 (2.743-10.900)** | **<0.001** | **2.469 (1.271-4.800)** | **0.008** |
| Low oxygen saturation (SpO2 < 90%) | 18/60 (30.0) | **0.535 (0.301-0.948)** | **0.032** | 0.785 (0.402-1.532) | 0.477 |
| Lower extremity edema (emergency department) | 5/61 (8.2) | 0.523 (0.204-1.338) | 0.176 | 0.524 (0.192-1.434) | 0.208 |
| Wheezing upon auscultation (emergency department) | 9/61 (14.8) | 0.516 (0.249-1.070) | 0.075 | 0.466 (0.204-1.066) | 0.071 |
| Reduced vigilance (GCS < 15) | 6/61 (9.8) | **0.366 (0.155-0.868)** | **0.022** | **0.316 (0.113-0.883)** | **0.028** |
| **Dysrhythmia (n = 38)** | | | | | |
| Hypotension (systolic blood pressure < 100 mmHg) | 9/38 (23.7) | **4.810 (2.133-10.849)** | **<0.001** | **5.018 (1.931-13.045)** | **0.001** |
| Tachycardia (heart rate > 100/minute) | 26/38 (68.4) | **3.109 (1.542-6.267)** | **0.002** | **4.511 (2.104-9.672)** | **<0.001** |
| Lower extremity edema (emergency department) | 11/38 (28.9) | **2.675 (1.282-5.581)** | **0.009** | **3.837 (1.677-8.782)** | **0.001** |
| Wheezing upon auscultation (emergency department) | 4/38 (10.5) | 0.353 (0.123-1.009) | 0.052 | 0.553 (0.176-1.738) | 0.311 |
| Wheezing upon auscultation (out-of-hospital) | 3/38 (7.9) | **0.230 (0.070-0.756)** | **0.016** | 0.409 (0.112-1.487) | 0.174 |
| Low oxygen saturation (SpO2 < 90%) | 8/38 (21.1) | **0.332 (0.150-0.735)** | **0.007** | **0.298 (0.124-0.717)** | **0.007** |
| Tachypnea (respiratory rate ≥ 22/minute) | 4/30 (13.3) | **0.239 (0.082-0.696)** | **0.009** | **0.283 (0.090-0.893)** | **0.031** |
| **Urinary tract infection (n = 35)** | | | | | |
| Reduced vigilance (GCS < 15) | 20/35 (57.1) | **5.343 (2.665-10.714)** | **<0.001** | **3.406 (1.552-7.477)** | **0.002** |
| Hypotension (systolic blood pressure < 100 mmHg) | 8/35 (22.9) | **4.495 (1.921-10.516)** | **0.001** | **2.946 (1.123-7.730)** | **0.023** |
| High body temperature (temperature ≥ 38 °C) | 11/25 (44.0) | **3.886 (1.697-8.897)** | **0.001** | **2.317 (1.005-5.339)** | **0.049** |
| Crackles upon auscultation (out-of-hospital) | 6/35 (17.1) | **2.064 (1.027-4.149)** | **0.042** | 1.609 (0.737-3.516) | 0.233 |
| Wheezing upon auscultation (out-of-hospital) | 5/35 (14.3) | 0.456 (0.174-1.194) | 0.110 | 0.853 (0.294-2.470) | 0.769 |
| Wheezing upon auscultation (emergency department) | 4/35 (11.4) | 0.389 (0.135-1.119) | 0.080 | 0.642 (0.204-2.023) | 0.449 |
| Lower extremity edema (emergency department) | 1/35 (2.9) | 0.172 (0.023-1.269) | 0.084 | 0.209 (0.027-1.591) | 0.131 |
| Tachycardia (heart rate > 100/minute) | 11/35 (31.4) | 0.605 (0.292-1.255) | 0.177 | **0.443 (0.203-0.969)** | **0.041** |
| **Hypertensive crisis (n = 33)** | | | | | |
| Crackles upon auscultation (emergency department) | 10/33 (30.3) | 1.755 (0.816-3.775) | 0.150 | 1.755 (0.816-3.775) | 0.150 |
| **Pulmonary embolism (n = 18)** | | | | | |
| Reported pain (NRS ≥ 1) | 7/13 (53.8) | 2.454 (0.809-7.444) | 0.113 | 0.977 (0.345-2.768) | 0.965 |
| Tachypnea (respiratory rate ≥ 22/minute) | 1/14 (7.1) | **0.123 (0.016-0.947)** | **0.044** | 0.271 (0.038-1.935) | 0.192 |
| Wheezing upon auscultation (out-of-hospital) | 1/18 (5.6) | 0.162 (0.021-1.223) | 0.078 | 0.207 (0.026-1.621) | 0.134 |
| Crackles upon auscultation (out-of-hospital) | 1/18 (5.6) | 0.170 (0.022-1.288) | 0.086 | 0.192 (0.025-1.494) | 0.115 |
| **Asthma (n = 15)** | | | | | |
| Wheezing upon auscultation (out-of-hospital) | 14/15 (93.3) | **42.644 (5.567-326.637)** | **<0.001** | **38.7886 (4.779-314.754)** | **0.001** |
| Tachycardia (heart rate > 100/minute) | 10/15 (66.7) | 2.760 (0.934-8.161) | 0.066 | 2.118 (0.671-6.682) | 0.201 |
| Tachypnea (respiratory rate ≥ 22/minute) | 7/9 (77.8) | **5.966 (1.227-29.009)** | **0.027** | 1.873 (0.502-6.987) | 0.349 |
| Wheezing upon auscultation (emergency department) | 7/15 (46.7) | **2.808 (1.003-7.860)** | **0.049** | 0.776 (0.253-2.384) | 0.658 |
| Crackles upon auscultation (out-of-hospital) | 1/15 (6.7) | 0.208 (0.027-1.592) | 0.131 | 0.201 (0.025-1.589) | 0.128 |

*Binary logistic regression: findings with p < 0.2 in univariable analysis are shown and included in multivariable analysis; significant findings (p < 0.05)* *are shaded grey;* ***OR:*** *odds ratio;* ***95% CI:*** *95% confidence interval of OR;* ***GCS:*** *Glasgow Coma Scale;* ***NRS:*** *Numeric Rating Scale;* ***SpO2:*** *peripheral oxygen saturation. The following variables were reviewed for associations with discharge diagnoses: out-of-hospital findings: systolic blood pressure < 100 mmHg, heart rate > 100/min, peripheral oxygen saturation < 90%, respiratory rate ≥ 22/min, body temperature ≥ 38 °C, body temperature ≤ 36 °C, Glasgow Coma Scale < 15, numeric rating scale ≥ 1, crackles upon auscultation, wheezing upon auscultation, emergency department findings: crackles upon auscultation, wheezing upon auscultation, silent lung upon auscultation, and lower extremity edema*.
