# Supplementary material for: Shedding light into the black box of out-of-hospital respiratory distress—A retrospective cohort analysis of discharge diagnoses, prehospital diagnostic accuracy, and predictors of mortality
Source: PLoS One. 2022 Aug 3;17(8):e0271982. doi: 10.1371/journal.pone.0271982 (PMC9348717; doi:10.1371/journal.pone.0271982)
Supplement: S3 Table — Proportion of initial out-of-hospital examination findings that were not documented in the PEMT protocol. (DOCX) [file pone.0271982.s003.docx]

**S3 Table. Parameters not documented by the PEMT.**

| **Examination findings** | **Not documented** |
| --- | --- |
| Glasgow Coma Scale (GCS), n (%) | 13/719 (1.8) |
| Systolic blood pressure, n (%) | 21/719 (2.9) |
| Diastolic blood pressure, n (%) | 47/719 (6.5) |
| Heart rate, n (%) | 9/719 (1.3) |
| Peripheral oxygen saturation (SpO2), n (%) | 5/719 (0.7) |
| Respiratory rate, n (%) | 188/719 (26.1) |
| Body temperature, n (%) | 254/719 (35.3) |
| Numeric rating scale, n (%) | 287/719 (39.9) |

*Proportion of initial out-of-hospital examination findings that were not documented in the PEMT protocol.*
