# Supplementary material for: Shedding light into the black box of out-of-hospital respiratory distress—A retrospective cohort analysis of discharge diagnoses, prehospital diagnostic accuracy, and predictors of mortality
Source: PLoS One. 2022 Aug 3;17(8):e0271982. doi: 10.1371/journal.pone.0271982 (PMC9348717; doi:10.1371/journal.pone.0271982)
Supplement: S4 Table — Binary logistic regression: the following variables were tested for their associations with hospital mortality using univariable analyses: age, sex, misdiagnosis by the PEMT, discharge diagnoses with n > 10, out-of-hospital findings: systolic blood pressure < 100 mmHg, heart rate > 100/min, peripheral oxygen saturation < 90%, respiratory rate ≥ 22/min, body temperature ≥ 38°C, body temperature ≤ 36°C, Glasgow Coma Scale < 15, numeric rating scale ≥ 1, crackles upon auscultation, wheezing upon auscultation, emergency department findings: crackles upon auscultation, wheezing upon auscultation, silent lung upon auscultation, and lower extremity edema. Any variables with p < 0.2 in the univariable analysis are shown in the table and included in multivariable analysis. Significant findings (p < 0.05) are shaded grey. (DOCX) [file pone.0271982.s004.docx]

**S4 Table. Predictors of hospital mortality.**

|  | **Present/**  **encounters in which data is available n/n (%)** | **Present/**  **deaths in which data is available n/n (%)** | **Univariable analysis** | | **Multivariable analysis using multiple imputations** | |
| --- | --- | --- | --- | --- | --- | --- |
|  |  |  | **OR (95% CI)** | **p value** | **OR (95% CI)** | **p value** |
| **Demographic factors** | | | | | | |
| Age by 1 year increase |  |  | **1.044 (1.026-1.062)** | **<0.001** | **1.031 (1.007-1.055)** | **0.011** |
| Age < 60 years | 170/718 (23.7) | 7/98 (7.1) | **0.216 (0.098-0.475)** | **<0.001** | 0.375 (0.133-1.047) | 0.061 |
| Age 60–69 years | 111/718 (15.5) | 10/98 (10.2) | 0.584 (0.293-1.162) | 0.125 | 0.496 (0.173-1.416) | 0.190 |
| Age 70–79 years | 190/718 (26.5) | 30/98 (30.6) | 1.268 (0.796-2.021) | 0.317 |  |  |
| Age 80–89 years | 197/718 (27.4) | 44/98 (44.9) | **2.487 (1.605-3.854)** | **<0.001** | **1.868 (1.033-3.378)** | **0.039** |
| Age ≥ 90 years | 50/718 (7.0) | 7/98 (7.1) | 1.032 (0.451-2.364) | 0.940 |  |  |
| Female sex | 335/715 (46.9) | 56/97 (57.7) | **1.660 (1.076-2.559)** | **0.022** | 1.730 (0.964-3.104) | 0.066 |
| **Pathological examination findings** | | | | | | |
| Reduced vigilance (GCS < 15) | 154/706 (21.8) | 53/96 (55.2) | **6.212 (3.939-9.795)** | **<0.001** | **2.583 (1.341-4.976)** | **0.005** |
| Low oxygen saturation (SpO2 < 90%) | 309/714 (43.3) | 70/99 (70.7) | **3.797 (2.392-6.029)** | **<0.001** | **2.231 (1.193-4.173)** | **0.012** |
| Low body temperature (temperature ≤ 36 °C) | 74/465 (15.9) | 18/78 (23.1) | 1.773 (0.975-3.225) | 0.060 | 1.762 (0.811-3.832) | 0.152 |
| Tachypnea (respiratory rate ≥ 22/minute) | 200/531 (37.7) | 35/74 (47.3) | 1.588 (0.968-2.605) | 0.067 | 1.578 (0.817-3.046) | 0.174 |
| Hypotension (systolic blood pressure < 100 mmHg) | 49/698 (7.0) | 14/91 (15.4) | **2.971 (1.530-5.772)** | **0.001** | 1.747 (0.687-4.440) | 0.241 |
| Tachycardia (heart rate > 100/minute) | 302/710 (42.5) | 49/98 (50.0) | 1.419 (0.925-2.176) | 0.109 | 1.417 (0.772-2.599) | 0.260 |
| Crackles upon auscultation (out-of-hospital) | 181/719 (25.2) | 37/99 (37.4) | **1.973 (1.261-3.087)** | **0.003** | 1.032 (0.551-1.933) | 0.921 |
| Reported pain (NRS ≥ 1) | 142/432 (32.9) | 6/52 (11.5) | **0.234 (0.097-0.562)** | **0.001** | 0.886 (0.497-1.581) | 0.683 |
| Wheezing upon auscultation (out-of-hospital) | 188/719 (26.1) | 14/99 (14.1) | **0.422 (0.234-0.763)** | **0.004** | 0.669 (0.290-1.540) | 0.344 |
| Lower extremity edema (emergency department) | 101/719 (14.0) | 8/99 (8.1) | 0.498 (0.234-1.061) | 0.071 | 0.588 (0.228-1.518) | 0.273 |
| High body temperature (temperature ≥ 38 °C) | 85/465 (18.3) | 10/78 (12.8) | 0.612 (0.301-1.244) | 0.175 | 0.452 (0.197-1.037) | 0.061 |
| Wheezing upon auscultation (emergency department) | 174/719 (24.2) | 9/99 (9.1) | **0.275 (0.136-0.559)** | **<0.001** | **0.362 (0.136-0.961)** | **0.041** |
| **Diagnostic accuracy by the PEMT** | | | | | | |
| Misdiagnosis by the PEMT | 209/694 (30.1) | 41/96 (42.7) | **1.908 (1.226-2.968)** | **0.004** | 1.313 (0.728-2.368) | 0.366 |
| **Discharge diagnoses** | | | | | | |
| Pneumonia | 142/719 (19.7) | 44/99 (44.4) | **4.261 (2.713-6.692)** | **<0.001** | **3.221 (1.562-6.644)** | **0.002** |
| COPD exacerbation | 138/719 (19.2) | 6/99 (6.1) | **0.239 (0.102-0.557)** | **0.001** | 0.745 (0.247-2.243) | 0.600 |

***Binary logistic regression:*** *the following variables were tested for their associations with hospital mortality using univariable analyses: age, sex, misdiagnosis by the PEMT, discharge diagnoses with n > 10, out-of-hospital findings: systolic blood pressure < 100 mmHg, heart rate > 100/min, peripheral oxygen saturation < 90%, respiratory rate ≥ 22/min, body temperature ≥ 38 °C, body temperature ≤ 36 °C, Glasgow Coma Scale < 15, numeric rating scale ≥ 1, crackles upon auscultation, wheezing upon auscultation, emergency department findings: crackles upon auscultation, wheezing upon auscultation, silent lung upon auscultation, and lower extremity edema.* Any *variables* *with p < 0.2 in the univariable analysis are shown in the table and included in multivariable analysis. Significant findings (p < 0.05)* *are shaded grey.*

***n:*** *group size of each factor;* ***OR:*** *odds ratio;* ***95% CI:*** *95% confidence interval of OR;* ***GCS:*** *Glasgow Coma Scale;* ***SpO2:*** *peripheral oxygen saturation;* ***NRS:*** *Numeric Rating Scale;* ***PEMT:*** *physician-staffed emergency medical team;* ***COPD:*** *chronic obstructive pulmonary disease.*
