# Supplementary material for: Shedding light into the black box of out-of-hospital respiratory distress—A retrospective cohort analysis of discharge diagnoses, prehospital diagnostic accuracy, and predictors of mortality
Source: PLoS One. 2022 Aug 3;17(8):e0271982. doi: 10.1371/journal.pone.0271982 (PMC9348717; doi:10.1371/journal.pone.0271982)
Supplement: S5 Table — (DOCX) [file pone.0271982.s005.docx]

**S5 Table. Characterization of the most frequent discharge diagnoses.**

***COPD:*** *chronic obstructive pulmonary disease;* ***GCS:*** *Glasgow Coma Scale.*
